# Exosomes: efficient macrophage-related immunomodulators in chronic lung diseases

**DOI:** 10.3389/fcell.2024.1271684

**Published:** 2024-04-09

**Authors:** Jianxiong Kang, Peiyan Hua, Xiaojing Wu, Bin Wang

**Affiliations:** ^1^ Department of Thoracic Surgery at The Second Hospital of Jilin University, Changchun, Jilin, China; ^2^ Department of Hepatology, The First Hospital of Jilin University, Changchun, Jilin, China

**Keywords:** exosomes, macrophage, immunomodulator, chronic lung disease, immune balance

## Abstract

Macrophages, the predominant immune cells in the lungs, play a pivotal role in maintaining the delicate balance of the pulmonary immune microenvironment. However, in chronic inflammatory lung diseases and lung cancer, macrophage phenotypes undergo distinct transitions, with M1-predominant macrophages promoting inflammatory damage and M2-predominant macrophages fostering cancer progression. Exosomes, as critical mediators of intercellular signaling and substance exchange, participate in pathological reshaping of macrophages during development of pulmonary inflammatory diseases and lung cancer. Specifically, in inflammatory lung diseases, exosomes promote the pro-inflammatory phenotype of macrophages, suppress the anti-inflammatory phenotype, and subsequently, exosomes released by reshaped macrophages further exacerbate inflammatory damage. In cancer, exosomes promote pro-tumor tumor-associated macrophages (TAMs); inhibit anti-tumor TAMs; and exosomes released by TAMs further enhance tumor proliferation, metastasis, and resistance to chemotherapy. Simultaneously, exosomes exhibit a dual role, holding the potential to transmit immune-modulating molecules and load therapeutic agents and offering prospects for restoring immune dysregulation in macrophages during chronic inflammatory lung diseases and lung cancer. In chronic inflammatory lung diseases, this is manifested by exosomes reshaping anti-inflammatory macrophages, inhibiting pro-inflammatory macrophages, and alleviating inflammatory damage post-reshaping. In lung cancer, exosomes reshape anti-tumor macrophages, inhibit pro-tumor macrophages, and reshaped macrophages secrete exosomes that suppress lung cancer development. Looking ahead, efficient and targeted exosome-based therapies may emerge as a promising direction for treatment of pulmonary diseases.

## 1 Introduction

Pulmonary macrophages, the most abundant immune cells in the lungs, can be classified into distinct subtypes based on their functions, phenotypes, and secretory profiles. These subtypes include M1, M2, and other subgroups of macrophages (Aegerter et al., 2022). M1 macrophages, also known as “classically activated macrophages,” secrete proinflammatory cytokines such as tumor necrosis factor-alpha (TNF-α), interleukin (IL)-1β, IL-6, IL-12. In addition, M1 macrophages present foreign pathogens, cell debris, aging cells, and tumor cell antigens and engulf them (Yunna et al., 2020). However, excess of pro-inflammatory cytokines leads to an uncontrolled inflammatory response, resulting in damage to bronchial epithelial and alveolar cells, mucus obstruction of airways which restricts ventilation, and excessive activation of fibroblasts and collagen deposition (Dong et al., 2022). M2 macrophages, also known as “alternatively activated macrophages,” secrete anti-inflammatory cytokines including transforming growth factor beta (TGF-β), IL-10, CC motif chemokine ligand (CCL)18, and CCL22, exerting immunomodulatory functions (Cheng et al., 2021). They can further be divided into M2a, M2b, M2c, and M2d subtypes. M2a macrophages are associated with allergic reactions and secrete pro-fibrotic factors necessary for tissue repair, while M2b macrophages secrete anti-inflammatory cytokines such as IL-10 and have the ability to recruit regulatory T cells to combat inflammation (Yang et al., 2019; Gopalakrishnan et al., 2022). M2c macrophages suppress immune responses and promote tissue repair (Junior et al., 2021). M2d macrophages, also known as tumor-associated macrophages (TAMs), suppress inflammatory responses and promote angiogenesis and tumor growth (Zhang and Sioud, 2023).

Macrophages undergo substantial phenotypic and functional changes upon activation triggered by injury, stimulation, or alterations in the lung microenvironment. They play a significant role in the pathophysiology of diverse lung diseases, including inflammatory lung diseases such as chronic obstructive pulmonary disease (COPD), acute lung injury or acute respiratory distress syndrome (ALI/ARDS), pulmonary fibrosis, asthma, and lung cancer (TJonck and Bain, 2023). Prolonged injury and irritation induce macrophage population dysregulation, amplify inflammation or induce abnormal proliferation of tumor cells, and disrupt the pulmonary microenvironment, thereby contributing to development of various lung diseases (Melo et al., 2021).

Exosomes are membranous particles synthesized and secreted by cells. They can carry various genetic materials and signaling molecules, thereby regulating the functional state of recipient cells (Xu et al., 2021). Abnormalities in the quantity, cargo, or surface proteins of exosomes can reflect the status of donor cells and the extracellular microenvironment. Notably, exosomes isolated from blood, human bronchoalveolar lavage fluid (BALF), or sputum have shown promise as diagnostic markers for various lung diseases, thereby attracting growing interest as both mechanistic players and potential therapeutic targets in the context of pulmonary disorders (Zhao et al., 2022).

This review focuses on the dual regulatory role of exosomes in chronic pulmonary diseases. First, it provides an overview of exosomes, including their definition, biogenesis, structure, and composition. Subsequently, from the perspective of crosstalk between exosomes and macrophages, this review analyzes the mechanisms underlying the disruption of immune balance in chronic pulmonary inflammatory diseases and lung cancer. Finally, it analyzes the dual-edged nature of exosomes, highlighting their reparative effects on immune dysregulation associated with macrophages in chronic pulmonary inflammatory diseases and lung cancer.

## 2 What are exosomes?

### 2.1 Exosomes

Extracellular vesicles are membrane-bound structures released by cells into the extracellular space. Based on particle size and biogenesis processes, extracellular vesicles can be broadly categorized into three main groups: exosomes (30–150 nm in diameter), microvesicles (100–1,000 nm in diameter), and apoptotic bodies (100–5,000 nm in diameter) (Liu and Wang, 2023). Apoptotic bodies are vesicular structures formed by membrane wrinkling and invagination during cell apoptosis, directly budding off. Microvesicles are also vesicular structures formed through direct budding and shedding (Kalluri and LeBleu, 2020). Compared to microvesicles and apoptotic bodies, exosomes exhibit a more uniform particle size (Mathivanan et al., 2010). Exosomes have a diverse range of functions, including intercellular communication (immune suppression, antigen presentation, transfer of signaling components, inflammation, tumor growth, metastasis, angiogenesis, and intercellular exchange of functional genetic information), cell adhesion, and coagulation (van der Pol et al., 2012). Some functions of microvesicles overlap with those of exosomes, such as cell adhesion and coagulation (Wolf, 1967; Merten et al., 1999).

### 2.2 Biogenesis of exosomes

**FIGURE 1:**
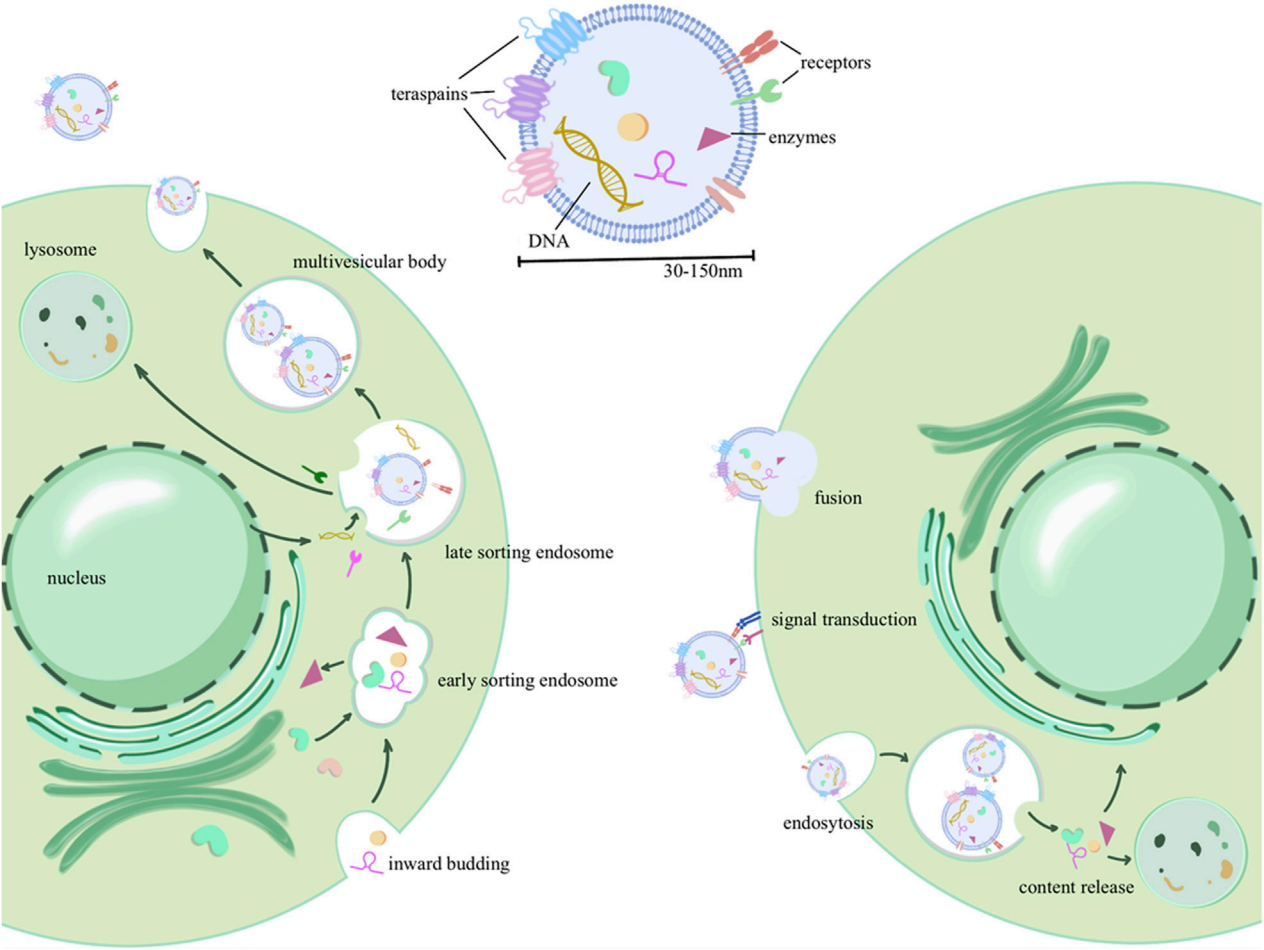
Exosome production process, contents, and delivery: the process initiates with invagination of the plasma membrane, forming early sorting endosomes (ESEs) that encapsulate extracellular components. These ESEs can fuse with intracellularly synthesized substances or exchange biomaterials, leading to the formation of late sorting endosomes (LSEs). Further maturation gives rise to intracellular multivesicular bodies (MVBs), from which intraluminal vesicles (ILVs) are selectively sorted and eventually released into the extracellular space as extracellular vesicles. In addition, three forms of extracellular vesicle uptake by recipient cells were described, including membrane fusion, receptor binding (signal transduction), and endocytosis.

Exosome biogenesis involves a complex series of membrane fusion and sorting processes to encapsulate and sort contents. Initially, there is an invagination of the cell membrane, encapsulating extracellular components, to form early sorting endosomes (ESEs). These ESEs can then fuse with each other or engage in material exchange with internally synthesized substances, resulting in the formation of late sorting endosomes (LSEs). Further sorting and fusion lead to the formation of multivesicular bodies (MVBs) within the cell. MVB biogenesis involves various mechanisms, including the endosomal sorting complex required for transport (ESCRT) machinery for the transport-associated intraluminal vesicle (ILV) sorting, triggered by ESCRT-I/II, as well as non-ESCRT mechanisms (Colombo et al., 2014). After maturation and sorting of intraluminal vesicles (ILVs, also known as exosomes) within MVBs, two potential fates emerge: MVBs can either fuse with the cell membrane, budding internally and releasing exosomes into the extracellular space, or they may undergo fusion with lysosomes for cargo degradation (Broad et al., 2023). Upon transport and docking at the plasma membrane, secretory MVBs bind to the cell’s inner membrane receptors (such as soluble N-ethylmaleimide-sensitive factor attachment protein receptor, SNARE), initiating release (Bebelman et al., 2018). As depicted in Figure 1, exosome formation involves multiple rounds of sorting and assembly, resulting in significant heterogeneity among exosomes. This heterogeneity is not only evident in their varying particle sizes but, more importantly, in their unique structures and compositions.

### 2.3 Structure and composition of exosomes

Generally, exosomes are primarily composed of a phospholipid membrane forming the outer shell, along with various cargo molecules such as RNA, DNA, and proteins (Mondal et al., 2023). The phospholipid bilayer of exosomes is relatively stable, allowing for long-distance transportation. In addition to maintaining the stability of the membrane skeleton, membrane proteins also participate in information exchange, such as selective binding with receptor cells. These membrane-encapsulated proteins can be transported to recipient cells, serving diverse functions such as enzymatic modification of downstream pathways or assisting in viral transmission by transporting viral antigens (Gurunathan et al., 2021). Some specific proteins are used for exosome identification, including tetraspanins CD63, CD9, and CD81; heat shock proteins HSP70 and HSP90; as well as apoptosis-linked gene 2-interacting protein X(ALIX) and tumor susceptibility gene 101(TSG101) (Pegtel and Gould, 2019). In addition to proteins, exosomes also carry various types of RNAs and DNA. Non-coding RNAs, in particular, can epigenetically modify gene expression frequencies in recipient cells, leading to altered functionality. The DNA and RNA in exosomes may carry genetic information from donor cells and viruses (Zaiets et al., 2023) and can serve as diagnostic markers for inflammation, cancer, and viral infections (Zhu et al., 2020).

## 3 The relationship between different subtypes of macrophages and inflammatory lung diseases and lung cancer

As mentioned earlier, macrophages are primarily divided into two subtypes, M1 and M2. M1 macrophages are typically associated with inflammation and immune response, while M2 macrophages are linked to anti-inflammatory and tissue repair processes (Aegerter et al., 2022). In inflammatory lung diseases such as asthma and COPD, as well as in lung cancer, the roles of M1 and M2 macrophages exhibit complex interrelationships.

In inflammatory lung diseases such as asthma and COPD, M1 macrophages are typically involved in the inflammatory process, producing inflammatory mediators and engulfing pathogens. The inflammatory response of M1 macrophages may be necessary in the early stages. However, excessive or prolonged inflammation may lead to lung tissue damage (Chen et al., 2020). M2 macrophages are generally associated with anti-inflammation and promotion of tissue repair processes. They may play a protective role in the later stages of inflammation, but excessive activated M2 macrophages can produce pro-fibrotic mediators, leading to sustained activation of fibroblasts, promoting myofibroblast proliferation, and facilitating collagen deposition (Zhang et al., 2018). In summary, maintaining a balance between M1 and M2 macrophages in inflammatory lung diseases can help resist the invasion of pulmonary pathogens, protect tissue cells from excessive inflammatory damage, and prevent fibrosis.

In lung cancer, tumor-associated macrophages (TAMs) are closely associated with tumor development, invasion, and metastasis, influencing the tumor microenvironment, immune suppression, and angiogenesis (Hu et al., 2022). TAMs are generally classified into two main subtypes: M1 macrophages and M2 macrophages. In the early stages of lung cancer, M1 macrophages may exhibit anti-tumor activity. They identify and destroy cancer cells, produce inflammatory mediators, activate immune cells, and participate in anti-tumor immune responses (Ma et al., 2010). M2 macrophages: as the tumor progresses, TAMs gradually transform into M2 macrophages, displaying immunosuppressive characteristics and promoting tumor growth. They release immunosuppressive molecules; inhibit anti-tumor immune responses; and simultaneously facilitate angiogenesis, invasion, and metastasis of tumor cells (Basak et al., 2023).

In summary, the roles of macrophages in inflammatory lung diseases and lung cancer may be complex and dynamic. The contradictory nature of these roles primarily depends on the specific functions of macrophages at different disease stages and their interactions with other immune cells and inflammatory mediators (Boutilier and Elsawa, 2021; Lee et al., 2021; Basak et al., 2023).

## 4 Exosomes are involved in macrophage-mediated formation of the pulmonary pathological microenvironment

**FIGURE 2:**
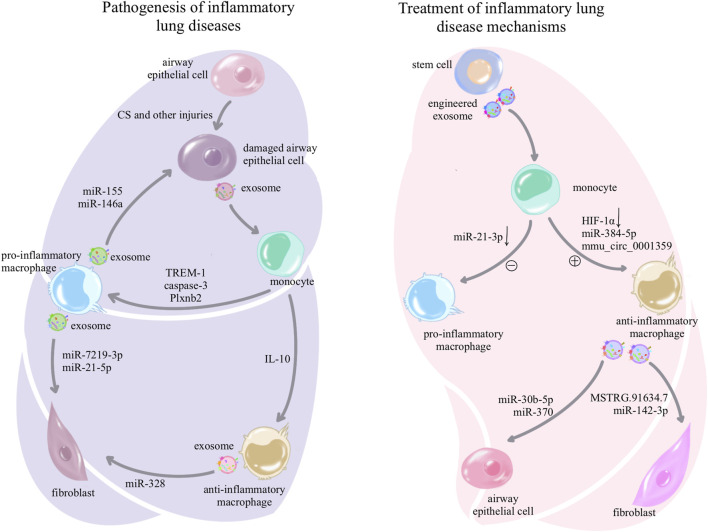
Pathogenesis and treatment of exosome-associated inflammatory lung disease: in inflammatory lung diseases, exosomes can be secreted by damaged airway epithelial cells, prompting macrophages to differentiate toward the pro-inflammatory phenotype and suppressing anti-inflammatory differentiation; while pro-inflammatory macrophages in turn use exosomes for further airway epithelial damage, fibroblast activation, and activation of other immune cells, forming a vicious circle to amplify inflammation and injury. However, the treatment of inflammatory lung diseases is delivered to macrophages through a variety of stem cell exosomes and synthetic drug-encapsulated exosomes, which induce anti-inflammatory differentiation and inhibit pro-inflammatory differentiation of macrophages, or the use of anti-inflammatory macrophage-secreted exosomes that act on other cells to mitigate the damage and destruction of lung tissue.

**FIGURE 3:**
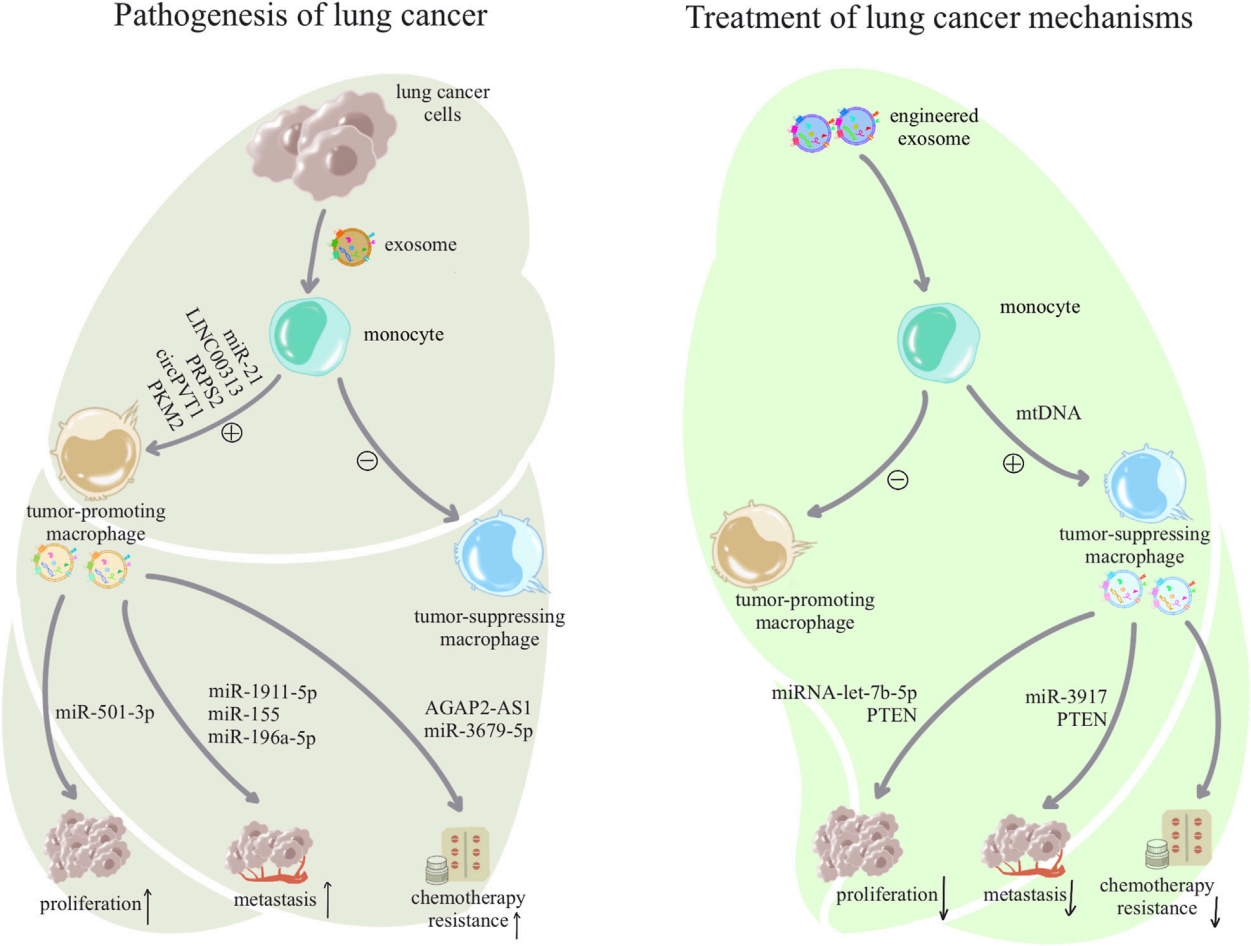
Pathogenesis and treatment of exosome-associated lung cancer: lung cancer cells use exosomes to alter macrophage subpopulations to form a tumor microenvironment, and dysregulated macrophages facilitate a variety of malignant behaviors such as growth, metastasis, and drug resistance of lung cancer cells through exosomes. In contrast, treatment of lung cancer can begin by awakening anti-tumor macrophages in macrophages and inhibiting tumor-assisting macrophages, and anti-tumor macrophages will also inhibit the proliferation and development of tumor cells through exosomes.

As crucial immune cells regulating the pulmonary microenvironment, macrophages play a pivotal role in promptly recognizing and engulfing invading pathogens, as well as senescent and aberrant cells entering the airways. They secrete cytokines to activate various immune cells, present antigens to initiate subsequent immune responses, and maintain a normal immune microenvironment in the lungs (Shotland et al., 2021). In this section, we elucidate the role of exosomes in macrophage-related immune dysregulation in inflammatory lung diseases and lung cancer, as shown in Table 1. In inflammatory lung diseases such as asthma, acute respiratory distress syndrome (ARDS), chronic obstructive pulmonary disease (COPD), and pulmonary fibrosis, exosomes reshape macrophages toward a pro-inflammatory phenotype, suppressing the anti-inflammatory phenotype. The reshaped macrophages release exosomes associated with inflammatory damage (Jiang et al., 2023), as shown in Figure 2. In lung cancer, exosomes reshape macrophages to promote a pro-tumorigenic phenotype while suppressing the anti-tumor phenotype. Tumor-associated macrophages release exosomes that further enhance tumor proliferation, metastasis, and resistance to chemotherapy (Liang et al., 2020), as shown in Figure 3.

**TABLE 1:** Exosomes are involved in macrophage-mediated formation of the pulmonary pathological microenvironment.

References	Disease	Donor cells	Cargo	Recipient cell	Experimental model	Functions
37,124,914 Jiang et al. (2023)	COPD	—	miR-7	Macrophages	CSE-induced mouse model of COPD	miR-7 from serum exosomes may exacerbate COPD by stimulating macrophage differentiation toward the M1 phenotype
32,259,794 Ye et al. (2020)	ARDS	Macrophages	Pro-inflammatory cytokines	Neutrophils	Lipopolysaccharide (LPS)-induced ARDS mouse model	Macrophages secrete exosomes containing multiple pro-inflammatory cytokines, which activate neutrophils to produce multiple pro-inflammatory cytokines
32,259,794 Ye et al. (2020)	ARDS	Neutrophils	IL-10	Macrophages	LPS-induced ARDS mouse model	Neutrophils produce exosomes of IL-10 that polarize macrophages into M2c
29,863,671 Yuan et al. (2018)	ARDS	Macrophages	miR-155 and miR-146a	Bronchial epithelial cells	LPS-induced ARDS mouse model	Exosomal delivery of miR-155 and miR-146a from macrophages disrupts the expression of tight junction proteins in bronchial epithelial cells
26,658,190 Moon et al. (2015)	ARDS	Lung epithelium cells	Cystatin-3	Macrophages	Mouse models exposed to room air (RA) or hyperoxia	Hyperoxia-induced, lung epithelium-derived cystatin-3-rich exosomes activate macrophages and mediate the inflammatory lung response
33,834,616 Qin et al. (2021)	Pulmonary fibrosis	Silica-exposed macrophages	—	Fibroblasts	Silicon dioxide-induced silicosis mouse model	Silica-exposed macrophage-derived exosomes induce endoplasmic reticulum stress in fibroblasts and promote lung fibrosis progression
34,483,252 (Sun et al., 2021a)	Idiopathic pulmonary fibrosis	Macrophages	Angiotensin II type 1 receptor	Fibroblasts	A mouse model of bleomycin (BLM)-induced pulmonary fibrosis	Macrophage exosomes transfer angiotensin II type 1 receptors to lung fibroblasts and mediate bleomycin-induced pulmonary fibrosis
35,689,956 Qian et al. (2022)	Pulmonary fibrosis	M2 macrophages	miR-129-5p	Fibroblasts	BLM-induced pulmonary fibrosis rat model	M2 macrophages can carry miR-129-5p into lung interstitial fibroblasts and cause fibroblast proliferation and pulmonary fibrosis
36,075,289 Niu et al. (2022)	Pulmonary fibrosis	Silica-exposed macrophages	miR-7219-3p	Fibroblasts	Silicon dioxide-induced silicosis mouse model	Silica-exposed macrophage exosomes overexpress miR-7219-3p, inhibit SPRY1, and activate ERK/MAPK phosphorylation to promote FMT, thereby promoting silica-induced pulmonary fibrosis
31,164,635 Yao et al. (2019)	Pulmonary fibrosis	M2 macrophages	miR-328	Fibroblasts	BLM-induced pulmonary fibrosis rat model	High expression of miR-328 by M2 macrophage-derived exosomes exacerbates pulmonary fibrosis by regulating FAM13A
23,414,598 Kulshreshtha et al. (2013)	Asthma	IL-13-activated epithelial cells	—	Macrophages	Mouse model of ovalbumin sensitization	IL-13-activated epithelial cell-derived exosomes can induce enhanced proliferation and chemotaxis of lung undifferentiated macrophages in asthma
34,040,396 Li et al. (2021a)	Asthma	Macrophages	miR-21-5p	Tracheal epithelial cells	Mouse model of ovalbumin sensitization	Macrophages translocate miR-21-5p to tracheal epithelial cells via exosomes, promoting EMT and airway remodeling
20,728,205 Esser et al. (2010)	Asthma	Macrophages and DCs	Leukotriene biosynthesis enzymes	Granulocytes	GM-CSF/IL4-induced macrophage models	Macrophage and dendritic cell exosomes contain enzymes that induce granulocyte migration to promote inflammation
34,414,666 Yu et al. (2021)	Asthma	Ovalbumin-treated airway epithelial cells	Plxnb2	Macrophages	A mouse model of ovalbumin induction	Ovalbumin-treated airway epithelium-derived exosomes are enriched with Plxnb2 protein and activate macrophage-mediated allergic inflammation via MMP14 cleavage of CD100
32,867,817 Liang et al. (2020)	Lung cancer	Lung cancer cells	TRIM59	Macrophages	Lewis lung cancer mouse model	Exosome TRIM59 promotes cancer progression by regulating ABHD5 proteasome degradation, promoting IL-1β secretion by macrophages, and activating the NLRP3 signaling pathway
34,559,989 Morrissey et al. (2021)	Lung cancer	Lung cancer cells	—	Macrophages	Lewis lung cancer mouse model	Induction of NF-kB activation via TLR2 leads to upregulation of PD-L1 by macrophages and polarization of tissue-resident macrophages to an immunosuppressive phenotype
36,270,983 Rao et al. (2022)	Lung cancer	Lung cancer cells	—	Macrophages	Mouse model of small cell lung cancer	Small cell lung cancer-derived exosomes induce macrophage differentiation to the M2 type
33,972,506 Zhang et al. (2021c)	Lung cancer	M2 macrophage	AGAP2-AS1	Lung cancer cells	A mouse model of radiation-resistant lung cancer cell induction	M2 macrophage-derived exosome AGAP2-AS1 enhances radioimmunity of lung cancer cells by decreasing miR-296 and elevating NOTCH2
32,456,301 Pritchard et al. (2020)	Lung cancer	Lung cancer cells	—	Macrophages	Lewis lung cancer mouse model	Lung tumor cell-derived exosomes promote M2 macrophage polarization
33,889,514 Li et al. (2021c)	Lung cancer	M2 macrophages	miR-155 and miR-196a-5p	Lung cancer cells	Mouse models of non-small cell lung cancer induction	M2 tumor-associated macrophages secrete the exosomes miR-155 and miR-196a-5p to promote non-small cell lung cancer metastasis
35,897,096 Jin and Yu (2022)	Lung cancer	Lung cancer cells	miR-21	Macrophages	Mouse models of non-small cell lung cancer induction	Non-small cell lung cancer cells secrete miR-21-rich exosomes that target IRF1 to promote macrophage M2 polarization in a hypoxic environment
36,660,623 Yan et al. (2022)	Lung cancer	Lung cancer cells	miR-146a	Macrophages	A cellular model of lung cancer cell exosomes co-cultured with macrophages	Exosomal miR-146a from non-small cell lung cancer cells inhibited TRAF-6 and IRAK-1 expression in macrophages, leading to inhibition of M1 polarization
35,980,503 Kong et al. (2022)	Lung cancer	Lung cancer cells	LINC00313	Macrophages	Mouse models of non-small cell lung cancer induction	Non-small cell lung cancer cell-derived exosomes LINC00313 upregulate macrophage STAT6 expression, leading to M2 macrophage differentiation
35,025,697 Liu et al. (2022b)	Lung cancer	Lung cancer cells	circPVT1	Macrophages	A cellular model of lung cancer cell exosomes co-cultured with macrophages	CircPVT1 in lung cancer exosomes induces macrophage polarization toward the M2 phenotype through the miR-124-3p/EZH2 axis and enhances proliferation, invasion, and migration of lung cancer cells
36,168,315 Yuan et al. (2022)	Lung cancer	Tumor-associated macrophages	—	Lung cancer cells	A cell model of co culturing extracellular vesicles of macrophages with lung adenocarcinoma cells	Tumor-associated macrophage-derived exosomes promote EGFR-TKI resistance in non-small cell lung cancer by regulating the AKT, ERK1/2 and STAT3 signaling pathways
35,818,293 Zhou et al. (2022)	Lung cancer	Lung cancer cells	PKM2	Macrophages	Mouse models of lung cancer induction	High expression of PKM2 in exosomes of non-small cell lung cancer cell origin induces M2 macrophage polarization via the AMPK pathway under hypoxic conditions
36,709,645 Chen et al. (2023)	Lung cancer	Irradiated lung cancer cells	miR-4655-5p	Macrophages	A cellular model of post-irradiation lung cancer cell exosomes co-cultured with macrophages	Irradiated cancer cell-derived exosomes enriched with miR-4655-5p inhibit MID1 and thus promote macrophage proliferation and M2 polarization
33,748,098 Wang et al. (2020)	Lung cancer	M2 macrophages	miR-3679-5p	Lung cancer cells	Mouse models of lung cancer induction	M2 macrophage-derived exosomes downregulate the expression of E3 ligase NEDD4L, leading to stabilization of c-Myc and elevated glycolysis, and then leads to chemotherapy resistance in cancer
34,251,965 Liu et al. (2022a)	Lung cancer	Lung cancer cells	PRPS2	Macrophages	A cellular model of macrophage exosomes co-cultured with lung cancer cells	In non-small cell lung cancer, tumor cell exosomes highly express PRPS2 to mediate macrophage M2 polarization to enhance resistance to cisplatin
36,604,626 Hu et al. (2023)	Lung cancer	Lung cancer cells	LINC00963	Macrophages	Mouse models of lung cancer induction	Lung adenocarcinoma cell exosomes induce M2 macrophage polarization through delivery of lncRNA LINC00963
36,730,375 Guan et al. (2023)	Lung cancer	M2 macrophages	miR-1911-5p	Lung cancer cells	A cellular model of macrophage exosomes co-cultured with lung cancer cells	M2 macrophage-derived exosome miR-1911-5p promotes migration and invasion of lung adenocarcinoma cells by inhibiting CELF2-activated ZBTB4
36,454,975 Qian et al. (2023)	Lung cancer	Lung cancer cells	Circ-ADRM1	Macrophages	Mouse models of lung cancer induction	Exosomes from lung adenocarcinoma cells induce M2 macrophage polarization through delivery of Circ-ADRM1
35,229,026 Chen et al. (2022a)	Lung cancer	Lung cancer cells	circSHKBP1	Macrophages	Mouse models of lung cancer induction	Non-small cell lung cancer exosomes induce macrophage recruitment and M2 macrophage polarization through delivery of circSHKBP1
37,143,656 Shao et al. (2023)	Lung cancer	Tumor-associated macrophages	miR-4443	T cells	A cellular model of macrophage exosomes co-cultured with lung cancer cells	Tumor-associated macrophages release exosomes that promote differentiation of naive T cells to Treg cells in malignant pleural effusions by delivering miR-4443
33,546,686 Lei et al. (2021)	Lung cancer	M2 macrophages	miR-501-3p	Lung cancer cells	A cellular model of macrophage exosomes co-cultured with lung cancer cells	The M2 macrophage-derived exosome miR-501-3p promotes lung cancer cell proliferation and invasion through downregulation of WDR82
35,168,607 Wan et al. (2022)	Lung cancer	M2 macrophages	MSTRG.292666.16	Lung cancer cells	Mouse models of lung cancer induction	M2-type macrophage-derived exosomes promote resistance to axitinib in NSCLC by regulating the MSTRG.292666.16/miR-6386-5p/MAPK8IP3 axis

### 4.1 Pulmonary inflammatory lung disease

#### 4.1.1 Exosomes reshape pro-inflammatory macrophages

In COPD, exosomes activate macrophages and induce differentiation toward the pro-inflammatory M1 type, leading to persistent inflammatory damage and formation of a pathological microenvironment in the lungs. Cigarette smoke exposure is an important risk factor for COPD. Wang et al. (2021) demonstrated that mouse airway epithelial cells treated with cigarette smoke extract (CSE) released exosomes expressing high levels of triggering receptor expressed on myeloid cells-1 (TREM-1), which influenced macrophages and promoted M1 polarization. Subsequently, the excessive inflammatory response mediated by M1 macrophages leads to bronchial wall thickening and alveolar structural damage. Additionally, in COPD mice, highly expressed miR-7 in serum exosomes acted on macrophages, regulated macrophage activation through proto-oncogene Pim-1(PIM1), and promoted differentiation into the M1 phenotype, exacerbating the inflammatory response (Jiang et al., 2023).

ALI/ARDS is characterized by direct or indirect lung injury and an excessive and uncontrolled systemic inflammatory response (Chen et al., 2020). The pathogenesis of ALI/ARDS is complex, involving both infectious and non-infectious factors. High oxygen injury is a significant non-infectious etiology, where pulmonary epithelial cells, upon exposure to high oxygen injury, secrete exosomes rich in caspase-3. These exosomes, through the ROCK1 pathway, activate macrophages, leading to an increased secretion of inflammatory proteins, such as macrophage inflammatory protein 2 (MIP-2), thereby exacerbating the inflammatory lung response and worsening lung injury (Moon et al., 2015).

In asthma, Persistent airway inflammation and inflammation-induced airway remodeling are critical factors. Damaged airway epithelial cells can activate monocytes–macrophages and various inflammatory cells through exosomes. For instance, IL-13-induced epithelial cells exacerbate asthma inflammation by inducing monocyte proliferation and chemotaxis via exosomal signaling. Conversely, inhibition of exosome production by GW4869 (exosome inhibitor) reduces monocyte proliferation and chemotaxis (Kulshreshtha et al., 2013). Additionally, exosomes derived from ovalbumin-primed airway epithelial cells are enriched in Plexin B2 (Plxnb2) protein and cleave CD100 via matrix metalloproteinase 14 (MMP14), leading to increased levels of soluble CD100, which activate macrophage-mediated allergic inflammation and enhance the recruitment of lung neutrophils, eosinophils, and dendritic cells (Yu et al., 2021).

#### 4.1.2 Exosomes inhibit anti-inflammatory macrophages

In inflammatory lung diseases, various etiological factors induce epithelial cell damage, promoting polarization of M1 macrophages, exacerbating inflammatory injury, and concurrently inhibiting the polarization of M2 macrophages. This imbalance between M1 and M2 cells further aggravates inflammation (Song et al., 2019). BEAS-2B cells (human bronchial epithelial cells) treated with CSE can reduce the polarization of M2 macrophages through modulated extracellular vesicles (He et al., 2019). Numerous studies have indicated that therapeutic exosomes can alleviate lung inflammation by promoting the polarization of M2 macrophages (Deng et al., 2020; Harrell et al., 2020). However, persistent inflammatory damage can also induce the polarization of M2 macrophages, leading to airway remodeling and fibrosis. For instance, in acute respiratory distress syndrome (ARDS), activated neutrophils release exosomes containing IL-10, polarizing macrophages into the M2c subtype, thereby inducing tissue remodeling and fibrosis after ALI (Ye et al., 2020).

#### 4.1.3 Exosomes from reshaped macrophages induce inflammatory injury

In ARDS, hyperactivated macrophages secrete exosomes that contribute to the activation of other inflammatory cells and damage airway epithelial cells, exacerbating inflammation and injury. For instance, macrophage-derived exosomes contain pro-inflammatory cytokines that activate neutrophils and enhance their inflammatory response, leading to uncontrolled inflammation in ARDS (45). Additionally, exosomes released by macrophages disrupt the expression of tight junction proteins in bronchial epithelial cells, compromising the structural barrier (Yuan et al., 2018).

In pulmonary fibrosis, dysregulated macrophage subpopulations secrete exosomes that contribute to fibroblast activation and collagen deposition. For example, in silicosis, macrophage-derived exosomes induced endoplasmic reticulum stress in fibroblasts, upregulated type I collagen and alpha-smooth muscle actin (α-SMA) expression, and exacerbated fibrosis progression (Qin et al., 2021). Moreover, silica-exposed macrophage exosomes express high levels of miR-7219-3p, which promotes fibroblast-to-myofibroblast trans-differentiation (FMT) and enhances silicosis-induced pulmonary fibrosis through Spouty1 (SPRY1) inhibition and extracellular signal-regulated protein kinase (ERK)/mitogen-activated protein kinase (MAPK) pathway activation (Niu et al., 2022). In a mouse model of bleomycin-induced fibrosis, macrophages exhibited increased levels of angiotensin II (Ang II) and angiotensin II type 1 receptor (AT1R). Furthermore, exosomes facilitated the transportation of Ang II from macrophages to fibroblasts, contributing to the progression of fibrosis (Sun et al., 2021). M2 macrophages can release pro-fibrotic factors and activate fibroblasts through the exosomal pathway. For example, M2 macrophage-derived exosomes containing high levels of miR-328 exacerbate pulmonary fibrosis by regulating Family with sequence similarity 13, member A (FAM13A) (Yao et al., 2019).

In asthma, macrophages are activated by exosomes and, in turn, activate and recruit more inflammatory cells through exosome-mediated signaling. For example, macrophages and dendritic cells secrete exosomes containing enzymes involved in leukotriene biosynthesis and hence promote granulocyte migration (Esser et al., 2010). Alveolar macrophages transport miR-21-5p via exosomes to tracheal epithelial cells, promoting epithelial–mesenchymal transition (EMT) and airway remodeling through the TGF-β1/Smad signaling pathway targeting Smad7(52).

### 4.2 Lung cancer

#### 4.2.1 Exosome remodeling pro-tumor TAMs

As mentioned Section 3, TAMs gradually shift toward an M2-dominant phenotype with the progression of tumors (Basak et al., 2023). M2-polarized macrophages, as an alternative activated form, have been implicated in various malignant processes, including promoting tumor cell proliferation and anti-apoptosis (Ye et al., 2018), metastasis (Zhang et al., 2021), increased vascular permeability and edema formation (Zhang et al., 2021), angiogenesis, and immune suppression (Mohapatra et al., 2021). Exosomes, as crucial mediators of communication between tumor cells and the immune microenvironment in the lungs, play a significant role in assisting tumor cells in reshaping M2-type TAMs and creating an immune microenvironment conducive to tumor growth (Qiu et al., 2022).

First, tumor cell-derived exosomes can deliver various non-coding RNAs, altering the epigenetics of macrophages and activating the polarization of M2-type macrophages. Examples include exosomes secreted by non-small cell lung cancer, which are rich in miR-21 (Jin and Yu, 2022), LINC00313 (Kong et al., 2022), and PRPS2 (Liu et al., 2022), promoting M2 polarization in macrophages. Liu et al. (2022) also found that exosomes from lung cancer contain circPVT1, which induces M2 polarization by inhibiting the expression of miR-124-3p in macrophages, leading to enhanced proliferation, invasion, and migration capabilities of lung cancer cells. Additionally, glycolysis is a crucial mechanism influencing macrophage polarization. For instance, pyruvate kinase M2 (PKM2) is a crucial molecule in macrophage metabolic adaptation, and tumor cells can activate PKM2 to regulate macrophage glycolysis, promoting the transition toward the M2 phenotype (Wu et al., 2022). Under hypoxic conditions, non-small cell lung cancer cells can directly secrete exosomes rich in PKM2 to induce M2 macrophage polarization through the AMPK pathway in macrophages (Zhou et al., 2022). Indirectly, through the delivery of exosomes, miR-1294 can upregulate PKM2 expression, promoting M2 macrophage polarization in an HIF-1α-dependent manner by regulating glycolysis (Chen et al., 2022). Furthermore, E-box-binding homeobox 1 (Zeb1) can induce the accumulation of M2-like tumor-associated macrophages (TAMs) through its involvement in glycolysis regulation (Jiang et al., 2022). Zeb1 can also induce the transcription of macrophage colony-stimulating factor (M-CSF) in cancer cells, leading to the secretion of M-CSF, driving M2-TAM polarization (Long et al., 2021). In lung adenocarcinoma, cancer cells can transfer LINC00963 through exosomes, stabilizing Zeb1 and stimulating M2 macrophage polarization (Hu et al., 2023). Additionally, matrix metalloproteinase 14 (MMP14), known as a target in various cancers (Liang et al., 2022), can not only promote tumor cell proliferation and migration but also facilitate the polarization of M2 macrophages. Exosomes from lung adenocarcinoma cells transmit Circ-ADRM1, recruiting USP12 to prevent ubiquitination of MMP14 protein and enhance MMP14 protein stability, thereby promoting M2-type macrophage polarization (Qian et al., 2023).

Additionally, TAMs exhibit a non-classical M2/M1 phenotype (Qian and Pollard, 2010). It is inappropriate to solely consider M2-polarized macrophages in TAMs as favorable for tumor growth and M1-polarized macrophages as inhibitory to tumor growth, as TAMs undergo reprogramming by tumor-derived exosomes. For instance, tumor-derived exosomes transfer tripartite motif-containing 59 (TRIM59) to macrophages, inducing the ubiquitination of abhydrolase domain-containing 5 (ABHD5), activating pro-tumor functions in macrophages. This activation occurs through the secretion of IL-1β, which activates the NLRP3 inflammasome signaling pathway, promoting the inflammatory microenvironment and cancer progression (Liang et al., 2020).

#### 4.2.2 Exosomes inhibit anti-tumor TAMs

Tumor-derived exosomes have been shown to exert immunosuppressive effects on macrophages, creating a microenvironment that facilitates tumor growth by suppressing “thermal immunity.” One mechanism involves the upregulation of programmed death ligand 1 (PD-L1), which promotes immunosuppression of macrophages. Tumor-derived exosomes activate NF-κB through Toll-like receptor-2 (TLR2), leading to the upregulation of PD-L1 expression. Moreover, increased glucose uptake and lactate conversion induced by these exosomes further enhance NF-κB activity, resulting in elevated PD-L1 levels. Consequently, tissue-resident macrophages are polarized toward an immunosuppressive phenotype (Morrissey et al., 2021). Furthermore, exosomes released by non-small cell lung cancer cells inhibit the expression of TNF receptor-associated factor 6 (TRAF6) and interleukin-1 receptor-associated kinase (IRAK1) in M0 macrophages, impairing M1 polarization and diminishing their ability to eliminate tumor cells (Yan et al., 2022). These findings underscore the role of tumor-derived exosomes in modulating macrophage function and shaping an immunosuppressive microenvironment that promotes tumor survival.

#### 4.2.3 TAM-derived exosomes promote tumor cell proliferation, migration, and chemotherapy resistance

As mentioned above, tumor-derived exosomes can reshape TAMs, particularly promoting the formation of immunosuppressive TAMs that dominate over anti-tumor TAMs (Qiu et al., 2022). TAMs, reshaped by tumor cells and the tumor microenvironment, secrete exosomes that promote tumor proliferation, invasion, metastasis, and resistance to chemotherapy or radiotherapy (Xu et al., 2022). This section primarily analyzes the impact of reshaped TAM-derived exosomes on tumor development.

First, TAMs promote the proliferation of lung cancer cells through exosomes. Specifically, exosomes derived from M2 macrophages have the ability to transport miR-501-3p to tumor cells. It has been discovered that WD repeat-containing 82 (WDR82) serves as the target gene for miR-501-3p, and its tumor-suppressive function has been demonstrated in rectal cancer (Liu et al., 2018; Li et al., 2021) Similarly, M2 macrophage-derived exosomes carrying miR-501-3p enhance lung cancer cell proliferation and invasion by downregulating WDR82 (76).

Second, TAMs also contribute to the invasion and metastasis of lung cancer cells through exosomes. For example, exosomes derived from M2 macrophages containing miR-1911-5p facilitate the migration and invasion of lung adenocarcinoma cells by silencing zinc finger and BTB domain-containing 4 (ZBTB4) (Guan et al., 2023), and ZBTB4 has been shown to regulate glycolipid metabolism and inhibit proliferation and invasion in malignancies such as pancreatic adenocarcinoma (Yang et al., 2023) and glioma (Dong et al., 2021). In lung cancer, ZBTB4 is also a target of CS-induced EMT (Cheng et al., 2021). Therefore, we speculated that M2 macrophage-derived exosomes reduce the expression of ZBTB4, thereby promoting the formation of a tumor-friendly microenvironment and facilitating tumor metastasis in advance. Additionally, another potential therapeutic target for inhibiting cancer cell invasion and EMT is RAS association domain family 4 (RASSF4) (Zhang et al., 2017). Exosomes carrying miR-155 and miR-196a-5p, secreted by M2 tumor-associated macrophages, negatively regulate the expression of RASSF4, thus promoting metastasis and EMT in non-small cell lung cancer (Li et al., 2021c).

Third, macrophages play a significant role in promoting chemotherapy resistance or radiation resistance in lung cancer cells through release of exosomes. For instance, the resistance of non-small cell lung cancer cells to epidermal growth factor receptor (EGFR)-targeted drugs could be attributed to the reactivation of AKT, ERK1/2, and signal transducer and activator of transcription 3 (STAT3) signaling pathways facilitated by exosomes released from macrophages (Yuan et al., 2022). Moreover, M2 macrophages can activate the MAPK signaling pathway via exosomes, leading to resistance against the EGFR-targeted drug osimertinib (Wan et al., 2022). Another factor contributing to drug resistance is heightened glycolysis. Increased expression of miR-3679-5p carried by M2 macrophage-derived exosomes downregulates the expression of E3 ligase NEDD4L (neural precursor cell-expressed developmentally downregulated gene 4-like), resulting in the stabilization of MYC proto-oncogene (c-Myc) and enhanced glycolysis. Enhanced glycolysis, in turn, leads to chemotherapy resistance in cancer cells (Wang et al., 2020). Apart from conferring chemotherapy resistance, exosomes can also induce radiation resistance. For instance, the M2 macrophage-derived exosome AGAP2 antisense RNA 1 (AGAP2-AS1) promotes the malignant phenotype of radiation-resistant cancer cells by reducing the levels of miR-296 and increasing the expression of notch homolog protein 2 (NOTCH2) (Rao et al., 2022).

Lastly, TAMs can modulate immune cells through exosomes, reshaping the immunosuppressive microenvironment and promoting tumor progression (Xu et al., 2022). For instance, TAMs with predominant M2 polarization secrete exosomes rich in miR-4443, facilitating the differentiation of naïve T cells into regulatory T (Treg) cells in malignant pleural effusion (Shao et al., 2023).

## 5 Exosomes target in macrophage-mediated repair of the pulmonary pathological microenvironment

As mentioned above, regulation of macrophages by exosomes plays a pathogenic role in promoting inflammation and tumor growth in inflammatory lung diseases and lung cancer. However, exosomes, as a double-edged sword, derived from stem cells or artificially manipulated exosomes, can also exert anti-inflammatory and anti-tumor effects (Gunassekaran et al., 2021; Wang et al., 2023). As shown in Table 2. This section will discuss the role of exosomes in reshaping macrophages toward an anti-inflammatory phenotype, inhibiting pro-inflammatory phenotypes, and reshaped macrophages release exosomes to alleviate inflammatory damage in inflammatory lung disease, as shown in Figure 2. Additionally, it will elaborate on how exosomes reshape anti-tumor macrophages, inhibit tumor-promoting macrophages, and how reshaped macrophages secrete exosomes to suppress tumor proliferation in lung cancer, as shown in Figure 3.

**TABLE 2:** Exosomes target in macrophage-mediated repair of the pulmonary pathological microenvironment.

References	Disease	Donor cells	Cargo	Recipient cell	Experimental model	Functions
35,924,248 Chen et al. (2022b)	COPD	Naringenin and CSE co-treated airway epithelial cells	miR-21-3p decreased	Macrophages	CSE-induced mouse and human cell line models	Naringenin and CSE co-treated reduction of miR-21-3p in bronchial epithelial cells secreting exosomes inhibits M1 macrophage polarization
35,016,678 Zhu et al. (2022)	COPD	ADSCs	—	Macrophages	CSE-induced mouse model of COPD	ADSC-derived exosomes effectively attenuate smoking-induced airway mucus overproduction, lung inflammation, and injury by inhibiting alveolar macrophage pyroptosis
30,981,817 He et al. (2019)	COPD	CSE-treated airway epithelial cells	miR-21 decreased	Macrophages	CSE-induced mouse model of COPD	CSE-treatment decreased exosomal miR-21 secretion by bronchial epithelial cells, thereby inhibiting M2 macrophage polarization and alleviating the EMT in the pathogenesis of COPD.
32,257,769 Harrell et al. (2020)	COPD	MSCs	—	Macrophages, neutrophils, and NK and T cells	CSE-induced mouse model of COPD; medication for COPD patients	MSC-derived exosome treatment attenuated the production of inflammatory cytokines in lung-infiltrating macrophages, neutrophils, NK, and T cells and attenuated the antigen-presenting properties of lung-infiltrating macrophages and DCs
33,753,901 Feng et al. (2021)	ARDS	Vascular endothelial cells and AEC II	CD31 and CD74 positive	Macrophages	Mouse models of ARDS induced by E. coli, LPS, and BLM	Vascular endothelial cells and AEC II secrete CD31 and CD74 positive exosomes, which regulate the immune homeostasis of alveolar macrophages
34,234,888 Liu et al. (2021a)	ARDS	BMSCs	miR-384-5p	Macrophages	Rat model of ALI induced by E. coli and LPS	miR-384-5p is enriched in BMSC-derived exosomes targeting beclin-1 to alleviate impaired autophagy in LPS-injured alveolar macrophages
32,433,208 Deng et al. (2020)	ARDS	Bone marrow stromal stem cells	—	Macrophages	LPS-induced ARDS mouse model and LPS-induced mouse alveolar macrophage cell line model	Bone marrow stromal stem cells inhibit M1 polarization and promote M2 polarization in mouse alveolar macrophages through inhibition of hypoxia-inducible factor 1 alpha
35,265,265 Deng et al. (2022)	ARDS	MSCs	—	Macrophages	LPS-induced ARDS mouse model and LPS-induced mouse alveolar macrophage cell line model	Exosomes from human MSCs can effectively downregulate sepsis-induced macrophage glycolysis and inflammation and ameliorate lung pathological injury
36,793,853 Feng et al. (2023)	ARDS	STIM-activating enhancer-positive type II AECs	—	Macrophages	BLM-induced injury model of mouse AEC-IIs	STIM-activating enhancer-positive type II AEC-derived exosomes regulate high Ca2+ responsiveness and long-term Ca2+ signaling, maintaining an M2-like immunophenotype, attenuating early acute injury, and preventing late fibrosis
37,285,229 Liu et al. (2023a)	ARDS	—	—	Macrophages	LPS-induced mouse model of ALI	The nanoplatform (termed D-SEL) is precisely delivered to macrophages to promote M2 macrophage polarization and alleviate acute inflammation in the lung
31,581,150 Mansouri et al. (2019)	Pulmonary fibrosis	BMSCs	—	Monocytes	BLM-induced pulmonary fibrosis model in mice	Human BMSC exosome therapy reprograms monocytes to a non-classical (Ly6Cneg) phenotype and attenuates pulmonary fibrosis and lung inflammation
28,853,608 Willis et al. (2018)	Bronchopulmonary dysplasia	MSCs	—	Macrophages	Mouse model of hyperoxia-induced lung dysplasia (BPD)	MSC-exosomes inhibit the pro-inflammatory “M1″ state and enhances the anti-inflammatory “M2-like” state, leading to improved lung function, reduced fibrosis, and pulmonary vascular remodeling
33,774,524 Sun et al. (2021b)	Pulmonary fibrosis	Fibroblasts	—	Macrophages	BLM-induced pulmonary fibrosis model in mice	A hybrid clodronate-loaded liposome and fibroblast-derived exosome (EL-CLD) delivery system loaded with the anti-fibrotic drug nintedanib effectively induces a diminished inflammatory response in macrophages
32,759,383 Guiot et al. (2020)	Idiopathic pulmonary fibrosis	Macrophages	miR-142-3p	Alveolar epithelial cells and fibroblasts	TGF-β-induced alveolar epithelial cell line and lung fibroblast cell line models	Macrophage-derived exosomes deliver miR-142-3p to alveolar epithelial cells and lung fibroblasts to counteract the progression of pulmonary fibrosis
34,435,585 Zhou et al. (2021)	Pulmonary fibrosis	Induced pluripotent stem cells	miR-302a-3p	M2 macrophages	BLM-induced pulmonary fibrosis model in mice	Induced pluripotent stem cell-derived exosomes suppress M2-type macrophages by delivering miR-302a-3p and silencing TET1, thereby attenuating lung fibrosis
36,309,172 Ban et al. (2023)	Pulmonary fibrosis	Macrophages	MSTRG.91634.7	Fibroblasts	Silicon dioxide-induced silicosis mouse model	Macrophage-derived exosomal lncRNA MSTRG.91634.7 targets PINK1 to inhibit fibroblast activation and limit silica-induced inflammation and fibrosis in mouse lungs
33,761,997 Dong et al. (2021b)	Asthma	Umbilical cord mesenchymal stem cells	—	Macrophages	Cellular models of LPS stimulation	Mesenchymal stem cell-derived exosomes modulate the inflammatory response by inhibiting TRAF1 remodeling of macrophage polarization, thereby ameliorating severe steroid-resistant asthma
35,500,231 Tang et al. (2022)	Asthma	M2 macrophages	miR-30b-5p	Airway epithelial cells	A mouse model of asthma induced by ovalbumin and aluminum hydroxide	Scorpion- and centipede-treated M2 macrophage exosomes carrying miR-30b-5p alleviate severe asthma by inhibiting airway epithelial cell apoptosis
33,994,863 Li et al. (2021d)	Asthma	M2 macrophages	miR-370	Airway smooth muscle cells	A mouse model of asthma induced by ovalbumin and aluminum hydroxide	M2 macrophage-derived exosomes carrying miR-370 alleviate asthma progression by inhibiting the FGF1/MAPK/STAT1 axis in airway smooth muscle cells
32,018,116 Shang et al. (2020)	Asthma	Adipose stem cells	mmu_circ_0001359	macrophages	Ovalbumin-induced mouse model of asthma	Adipose stem cell-derived exosomes enriched with mmu_circ_0001359 attenuate airway remodeling by promoting M2-like macrophages
33,360,827 Ren et al. (2021)	Asthma	MSCs	—	Macrophages	Ovalbumin-induced mouse model of asthma	Intranasal delivery of MSC-derived exosomes expands the proportion of IL-10-producing pulmonary interstitial macrophages in the lung and thus contributes to the prevention of allergic asthma
32,272,830 Li et al. (2020)	Lung cancer	Cisplatin-loaded M1 macrophages	Cisplatin	Lung cancer cells	Mouse models of lung cancer induction	M1 macrophage secretory exosome delivery system loaded with cisplatin inhibits proliferation and induces apoptosis in mouse Lewis lung cancer
36,054,073 Cui et al. (2022)	Lung cancer	MDA-MB-231 cells	—	Macrophages	Mouse models of lung cancer induction	Lung-specific exosome treatment combined with CD47 blockers and cisplatin enhances the phagocytic activity of macrophages while increasing T-cell proliferation
30,842,627 Kim et al. (2019)	Lung cancer	Macrophages exposed to apoptotic lung cancer cells	PTEN	Lung cancer cells	Mouse models of lung cancer induction	PTEN inhibits EMT and counteracts cancer progression and lung metastasis
28,982,587 Kim et al. (2018)	Lung cancer	Macrophages	Paclitaxel	Lung cancer cells	Mouse models of lung cancer induction	Macrophage-derived exosomes loaded with PTX represent a novel nano-agent that shows high anticancer efficacy in a mouse model of lung metastasis
36,643,646 Peng et al. (2023)	Lung cancer	M1 macrophages	miRNA-let-7b-5p	Lung cancer cells	A cellular model of macrophage exosomes co-cultured with lung cancer cells	M1 macrophage exosomes regulate the GNG5 signaling pathway by delivering miRNA-let-7b-5p to inhibit cancer cell proliferation and suppress the anti-apoptotic ability of cancer cells
34,195,198 Liu et al. (2021b)	Lung cancer	Lung cancer cells	miR-770	Macrophages	Mouse models of lung cancer induction	Tumor cell-derived exosome miR-770 inhibits non-small cell lung cancer invasion by targeting MAP3K1 to inhibit M2 macrophage polarization
36,261,031 Song et al. (2023)	Lung cancer	M2 macrophages	miR-3917	Lung cancer cells	Mouse models of lung cancer induction	M2 macrophages secrete exosomes that deliver miR-3917 and target G protein-coupled receptor kinase 6 to inhibit proliferation, migration, and invasion of H1299 cells
36,759,822 Lin et al. (2023)	Lung cancer	Lung cancer cells	CRV	Lung cancer cells and macrophages	Mouse models of lung cancer induction	CRV are constructed into cancer cell-derived exosomes to eliminate cancer cells and tumor-associated macrophages and reshape the tumor environment for effective cancer therapy
36,658,634 Zhang et al. (2023)	Lung cancer	—	—	M2 macrophages	Mouse models of lung cancer induction	Engineered exosomes targeting M2 macrophages inhibit PI-3 kinase γ expression and induce polarization of TAMs to M1 *in vitro* and *in vivo*, leading to increased T lymphocyte infiltration
36,879,291 Liu et al. (2023b)	Lung cancer	—	mtDNA	Macrophages	Mouse models of lung cancer induction	Induce the cGAS-STING pathway, drive the transition of pro-tumor macrophages to an anti-tumor phenotype, and enhance the efficacy of PD-L1 inhibitors

### 5.1 Inflammatory lung disease

#### 5.1.1 Exosomes reshape anti-inflammatory macrophages

In COPD, exosomes derived from stem cells can repair macrophage immune dysregulation and alleviate inflammatory damage. For instance, exosomes derived from mesenchymal stem cells (MSCs) have been found to interact with alveolar macrophages, inhibiting apoptosis and effectively mitigating persistent airway inflammation induced by cigarette smoke exposure (Zhu et al., 2022). Moreover, exosomes derived from MSCs facilitate the expansion of M2 macrophages and enhance the secretion of IL-10. This, in turn, induces the expansion of regulatory dendritic cells (DCs) and regulatory T cells, resulting in alterations to the immune microenvironment within the airways and consequently reducing chronic airway inflammation (Harrell et al., 2020).

In ARDS, M2 macrophages play a critical role in tissue repair and reducing inflammatory damage. Therefore, various engineered exosomes or stem cell-derived exosomes can alleviate inflammation in the acute phase of ARDS by promoting M2 macrophage polarization. For instance, an inhalable biomimetic sustained drug release nanoplatform, known as D-SEL, consisting of a combination of serum exosomes and liposomes encapsulating methylprednisolone succinate (MPS), can be precisely delivered to macrophages to promote M2 macrophage polarization and alleviate acute lung inflammation (Liu et al., 2023). Exosomes released by bone marrow mesenchymal stem cells (BMSCs) downregulate glycolysis by inhibiting HIF-1α, thereby promoting M2 polarization and attenuating sepsis-induced lung injury. These exosomes possess potent immunomodulatory and immunosuppressive properties (Deng et al., 2020). Exosomes derived from STIM-activating enhancer-positive type II alveolar epithelial cells (AEC II) regulate high Ca2+ responsiveness and long-term Ca2+ signaling, thereby maintaining an M2-like immune phenotype and metabolic selection. This modulation helps attenuate early acute injury and helps prevent late-stage fibrosis (Feng et al., 2023). Additionally, macrophages can be classified based on Ly6C into Ly6Clow, Ly6Chi, and Ly6C- macrophages. Exosomes derived from endothelial cells (EnCs) and AEC II suppress the expression of regulator of G protein signaling-1 (RGS1) in macrophages. RGS1 regulates macrophage Ca2+-dependent immune responses and modulates the recruitment of macrophages with different immune phenotypes during lung infection. Adjusting the pro-inflammatory or anti-fibrotic phenotype of Ly6C- macrophages promotes an increase in anti-inflammatory cytokines and tissue repair or fibrotic factors (Feng et al., 2021). Moreover, reducing macrophage autophagy also alleviates the progression of ARDS. BMSC-derived exosomes enriched in miR-384-5p target beclin-1 to alleviate autophagic stress in lipopolysaccharide (LPS)-injured alveolar macrophages and thereby alleviate inflammation (Liu et al., 2021).

In asthma, various stem cell-derived exosomes can promote M2 macrophage polarization to attenuate the inflammatory response and airway remodeling. For instance, exosomes derived from MSCs inhibit tumor necrosis factor receptor-associated factor 1 (TRAF1)-mediated macrophage polarization and promote M2 polarization, thereby modulating the inflammatory response and ameliorating severe steroid-resistant asthma (Dong et al., 2021). Exosomes derived from ADSCs modified with mmu_circ_0001359 absorb miR-183-5p to enhance forkhead box transcription factor 1 (FOXO1) signaling-mediated activation of M2 macrophages for treatment of asthma (Shang et al., 2020). Furthermore, intranasal delivery of mesenchymal stem cell-derived exosomes increases the proportion of IL-10-producing interstitial macrophages in the lungs, exerting anti-inflammatory effects and effectively treating allergic asthma (Ren et al., 2021).

#### 5.1.2 Exosomes inhibit pro-inflammatory macrophages

In COPD, the inhibition of M1 macrophage polarization has been shown to effectively reduce inflammatory infiltration. For instance, the co-treatment of bronchial epithelial cells with naringenin and CSE resulted in the secretion of exosomes with reduced levels of miR-21-3p, which targets phosphatase and tensin homolog (PTEN)/AKT, leading to inhibition of M1 macrophage polarization. This treatment also resulted in decreased secretion of TNF-α, IL-6, IL-1β, inducible nitric oxide synthase (iNOS), and IL-12 (98). Furthermore, the administration of exosomes derived from MSCs attenuated the production of inflammatory cytokines in lung-infiltrating macrophages, neutrophils, natural killer cells, and natural killer T cells, reducing the antigen-presenting capacity of lung-infiltrating macrophages and dendritic cells (Harrell et al., 2020). M2 macrophages can secrete anti-inflammatory cytokines but exacerbate EMT. Treatment with CSE resulted in a decrease in secretion of exosomal miR-21 by bronchial epithelial cells, thereby inhibiting M2 macrophage polarization and alleviating the pathogenesis of EMT in COPD (99).

In ARDS, exosomes derived from various stem cells exhibit the ability to inhibit pro-inflammatory macrophages or suppress the expression of pro-inflammatory factors, thereby mitigating early acute inflammation and reducing mortality. For instance, exosomes derived from human MSCs effectively downregulate macrophage glycolysis and the expression of pro-inflammatory factors induced by sepsis, leading to the amelioration of pulmonary pathological injury (Deng et al., 2022). As mentioned earlier, BMSCs promote M2 polarization by releasing exosomes that downregulate glycolysis and also exhibit inhibitory effects on M1 polarization. This modulation suppresses pro-inflammatory cytokines and prevents the escalation of the inflammatory response (Deng et al., 2020). Moreover, the previously mentioned D-SEL also promotes M2 polarization. Additionally, localized and sustained release of DNase I degrades dysregulated neutrophil extracellular traps (NETs), inhibiting neutrophil activation and the formation of a mucus-clogged microenvironment. This process further suppresses the recruitment of pro-inflammatory macrophages and reinforces M2 polarization (Liu et al., 2023).

In pulmonary fibrosis, exosomes derived from MSCs have the ability to suppress pro-inflammatory macrophages, resulting in the amelioration of chronic inflammation and pulmonary fibrosis. For instance, exosomes derived from human BMSCs suppress the pro-inflammatory monocyte phenotype and shift the distribution of pulmonary classical and non-classical monocytes toward that observed in control mice, including alveolar macrophages (Mansouri et al., 2019). MSC exosomes regulate and inhibit pro-inflammatory M1 alveolar macrophages both *in vitro* and *in vivo*, leading to improved lung function, reduced fibrosis, pulmonary vascular remodeling, and amelioration of pulmonary hypertension (Willis et al., 2018). A delivery system combining clodronate-loaded liposomes with fibroblast-derived exosomes (EL-CLD), loaded with the anti-fibrotic drug nintedanib, effectively induces a diminished inflammatory response in macrophages, providing a potential therapeutic approach for pulmonary fibrosis (Sun et al., 2021). Additionally, exosomes derived from induced pluripotent stem cells (iPSCs) suppress M2-type macrophages by delivering miR-302a-3p and silencing ten-eleven translocation 1 (TET1), thereby attenuating pulmonary fibrosis (Zhou et al., 2021).

In asthma, inhibiting the inflammatory response of macrophages can significantly alleviate disease progression and impaired ventilation. For example, exosomes derived from mesenchymal stem cells inhibit TRAF1 to remodel macrophage polarization and suppress M1 polarization, thereby modulating the inflammatory response and ameliorating severe steroid-resistant asthma (Dong et al., 2021).

#### 5.1.3 Exosomes from anti-inflammatory macrophages reduce inflammatory damage

In pulmonary fibrosis, exosomes derived from macrophages play a crucial role in targeting and inhibiting fibroblast activation and collagen deposition, thereby attenuating the exacerbation of fibrosis and impaired ventilation. For instance, macrophage-derived exosomes deliver miR-142-3p to alveolar epithelial cells and lung fibroblasts, effectively countering the progression of pulmonary fibrosis (Guiot et al., 2020). Moreover, macrophage-derived exosomes containing the lncRNA MSTRG.91634.7 target PTEN-induced putative kinase 1 (PINK1) to inhibit fibroblast activation, thereby limiting silica-induced lung inflammation and fibrosis in mice (Ban et al., 2023).

In asthma, exosomes derived from M2 macrophages have the ability to selectively target and act on airway epithelial cells and airway smooth muscle cells, thereby alleviating inflammation and fibrosis progression in asthma. For instance, M2 macrophage exosomes treated with the scorpion and centipede contain miR-30b-5p, which mitigates severe asthma by inhibiting apoptosis in airway epithelial cells (Tang et al., 2022). Additionally, M2 macrophage-derived exosomes carrying miR-370 alleviate asthma progression by suppressing the fibroblast growth factor-1 (FGF1) 1/MAPK/STAT1 axis in airway smooth muscle cells, resulting in the inhibition of abnormal proliferation, invasion, and the production of fibrosis-related proteins (Li et al., 2021).

### 5.2 Lung cancer

#### 5.2.1 Exosomes reshape anti-tumor TAMs

Synthetic exosomes can activate tumor-killing macrophages. For instance, the macrophage phosphoinositide 3-kinase gamma (PI3Kγ) is a crucial target for stimulating macrophage immunity and inhibiting tumor growth (Kaneda et al., 2016). This combination of gene editing technology with specific exosomes significantly improves targeting specificity and compatibility while reducing off-target effects and has been applied to various diseases. These exosomes specifically target and inhibit the expression of PI3Kγ in TAMs, leading to M1 polarization and reshaping of the tumor microenvironment, ultimately impeding tumor growth (Zhang et al., 2023). Drug delivery with engineered exosomes also enhances targeted drug delivery and relieves tumor inhibition of macrophages. For example, lung-specific exosomes combined with CD47 blockers and cisplatin treatment enhance macrophage phagocytic activity and promote T cell proliferation (Cui et al., 2022). In addition to the aforementioned engineered exosomes, plant-derived exosomes also exhibit the ability to activate tumor-killing macrophages. Nanovesicles derived from *Artemisia* species carrying plant-derived mitochondrial DNA (mtDNA) induce the cGAS (cyclic GMP–AMP synthase)–STING (stimulator of interferon genes) pathway, thereby promoting the transition of pro-tumor macrophages to an anti-tumor phenotype and enhancing the efficacy of PD-L1 inhibitors (Liu et al., 2023). Although traditional Chinese medicine offers numerous herbal treatments for tumors, oral absorption of these remedies is often inefficient and slow. However, research on plant-derived exosomes remains limited, holding the potential for unforeseen therapeutic benefits in tumor treatment.

#### 5.2.2 Exosomes inhibit pro-tumor TAMs

Interestingly, some tumor-derived exosomes have the ability to hinder their own proliferation and invasion by inhibiting macrophages. For example, cancer cell-derived exosomes engineered with tumor-associated antigens can inhibit M2 tumor-associated macrophages and myeloid-derived suppressor cells (MDSCs), while increasing CD8- and CD4-positive T cells. This leads to a remodeling of the tumor environment, enhancing the efficacy of cancer therapy (Lin et al., 2023). Additionally, tumor cell-derived exosomes containing miR-770 can impede M2 macrophage polarization by targeting MAP3K1, thereby suppressing invasion in non-small cell lung cancer (Liu et al., 2021). These oncogenic exosomes limit their own development by suppressing pro-tumor macrophage polarization. We speculate that this may involve a regulatory mechanism within the tumor itself, employing a negative feedback regulation to restrict growth for improved vascularization or to extend the latency period, thus favoring the long-term survival of cancer cells. Similarly, the hepatitis B virus can also exert negative regulation on its own replication through exosomes, enabling a more insidious replication to evade complete elimination by immune cells. Hepatitis B-infected hepatocytes release exosomes containing HBV-miR-3, which stimulate macrophages to secrete IL-6, consequently limiting HBV replication (Gan et al., 2022). Moreover, the target molecules involved in promoting macrophage activation and polarization, as mentioned above, can be targeted to inhibit the formation of an unfavorable tumor microenvironment and suppress tumor progression.

#### 5.2.3 Exosomes from anti-tumor TAMs inhibit tumor growth

First, exosomes derived from anti-tumor tumor-associated macrophages (TAMs) can inhibit cancer cell proliferation and metastasis. For instance, M1 macrophages release exosomes containing miRNA-let-7b-5p, which regulate the G protein subunit gamma 5 (GNG5) signaling pathway, suppressing cancer cell proliferation and inhibiting their anti-apoptotic ability (Peng et al., 2023). Cancer apoptosis cells irradiated with ultraviolet light, when co-cultured with macrophages, stimulate the production of exosomes rich in PTEN. These exosomes inhibit epithelial–mesenchymal transition (EMT), thereby limiting cancer progression and lung metastasis (Kim et al., 2019).

Additionally, exosomes from anti-tumor TAMs themselves exhibit anti-tumor effects. After artificial intervention to load drugs into them, their anti-cancer effects become more significant and highly specific. For example, exosomes derived from macrophages engineered to carry the anti-cancer drug paclitaxel (PTX) can be modified by incorporating aminoethyl anisamide (AA), a ligand specific to cancer sigma receptors, to enhance the targeting efficiency. Furthermore, adding polyethylene glycol (PEG) to these exosomes helps reduce immunogenicity and prolong circulation time. The resulting AA-PEG-modified exosomes loaded with PTX (AA-PEG-exoPTX) can selectively accumulate in cancer cells, significantly improving prognosis (Kim et al., 2018). A delivery system utilizing exosomes from M1 macrophages loaded with cisplatin effectively inhibits the proliferation of Lewis lung cancer cells in mice and induces apoptosis (Li et al., 2020).

Interestingly, M2 macrophages and their secreted exosomes are typically associated with promoting tumor behavior, but in some cancer cell lines, they can also exhibit anti-proliferative effects. For example, M2 macrophages release exosomes carrying miR-3917, which targets G protein-coupled receptor kinase 6 and inhibits proliferation, migration, and invasion of H1299 cells, while showing the opposite effect in A549 cells (cancer cell line) (Song et al., 2023). This difference may be attributed to variations in the expression frequency of miR-3917 downstream genes in different cell lines, providing new clues for the role of M2 macrophages in tumors. As shown in Table 2, extracellular vesicles target macrophages, mediating the repair of the pathological microenvironment in the lungs.

## 6 Conclusion and future directions

Macrophages represent the predominant immune cell population, participating in nearly all physiological and pathological processes in the lungs. Exosomes, as carriers for cellular material and information exchange, play a crucial role in regulating lung macrophages and reshaping the immune balance of the pulmonary microenvironment. In inflammatory lung diseases and lung cancer, exosomes derived from damaged airway epithelial cells, activated neutrophils, and tumor cells reshape the phenotype of macrophages. The reshaped macrophages exert pro-inflammatory and pro-tumor effects through exosome-mediated mechanisms, contributing to severe damage and tumor growth. Conversely, exosomes derived from stem cells or engineered exosomes reshape macrophage phenotypes and exhibit anti-inflammatory and anti-tumor effects through exosome-mediated mechanisms.

For future exosome-based therapies targeting inflammatory lung diseases and lung cancer, the pivotal directions of development may revolve around enhancing the specificity and efficiency. Specificity can be enhanced by constructing various target cell receptors and ligands on the exosome membrane surface, facilitating specific binding to target cells. Efficiency can be improved by exosomes derived from the same cell type for drug delivery, promoting enhanced “homing” compared to other drug carriers and exosomes derived from the deferent cell type, thus increasing uptake efficiency. Additionally, loading exosomes with tumor antigens can specifically enhance the antigen presentation ability of immune cells against tumor cells, efficiently activating endogenous “hot immune responses” to eliminate tumor cells. However, due to the complex composition and high heterogeneity of exosomes, challenges persist in achieving both specificity and efficiency.
